# AIDS Cholangiopathy Secondary to Cytomegalovirus as Possible Unmasking Immune Reconstitution Inflammatory Syndrome in Human Immunodeficiency Virus-Infected Individual: Case Report and Review of the Literature

**DOI:** 10.1155/2018/1891030

**Published:** 2018-07-26

**Authors:** Moiz Topiwala, Nachiket Dubale, Mahender K. Medisetty, Sunil Gaikwad, Divya Patel, Sanjay Pujari

**Affiliations:** ^1^Department of Internal Medicine, Poona Hospital and Research Centre, Pune, India; ^2^Department of Gastroenterology, Poona Hospital and Research Centre, Pune, India; ^3^Institute of Infectious Diseases, Pune, India; ^4^Department of Pathology, Poona Hospital and Research Centre, Pune, India

## Abstract

We present the first case report of cytomegalovirus (CMV) cholangiopathy as possible immune reconstitution inflammatory syndrome (IRIS) in a human immunodeficiency virus (HIV)-infected individual, within two months of starting effective HAART. The patient presented with abdominal pain, nausea, vomiting, decreased appetite, and jaundice. The patient was diagnosed on ERCP as AIDS cholangiopathy, and biopsy of the ampulla showed acute inflammation with CMV inclusion bodies. The patient underwent sphincterotomy with CBD stenting and HAART continued without use of ganciclovir or valganciclovir. On follow-up, the patient achieved clinical and histopathological cure, which was demonstrated on repeat ampullary biopsy.

## 1. Introduction

Acquired immune deficiency syndrome (AIDS) cholangiopathy is a biliary obstruction secondary to biliary tract strictures caused by various opportunistic infections [1]. Although common before the advent of highly active antiretroviral therapy (HAART), its incidence had decreased. It is usually seen amongst patients with CD4 count of <100/*μ*L. The common pathogen implicated is *Cryptosporidium parvum*, while others include *Cytomegalovirus* (CMV), *Microsporidium*, and *Cyclospora* [1, 2].

AIDS cholangiopathy is suspected when patients with advanced human immunodeficiency virus (HIV) infection present with right upper quadrant abdominal pain, diarrhoea, jaundice, and fever [3]. However, we describe here an HIV-infected individual who was diagnosed with AIDS cholangiopathy due to CMV most likely as an unmasking immune reconstitution inflammatory syndrome (IRIS).

## 2. Case Report

A 41-year-old HIV-1-infected male farmer presented with a five-day history of right hypochondriac abdominal pain associated with decreased appetite, nausea, vomiting, and two days of yellowish discolouration of sclera, with no history of fever, diarrhoea, blurring of vision, and odynophagia or dysphagia.

He presented to us for the first time in September 2016, when he was diagnosed with pneumocystis jiroveci pneumonitis (PJP) on transbronchial lung biopsy and successfully treated with co-trimoxazole and prednisolone. This biopsy did not reveal CMV inclusion bodies. His CD4 count was 109/*μ*L and HIV-1 viral load was 4.89 log copies/ml.

He was diagnosed with HIV-1 infection in 2000 and was treated by another physician, for which he was initiated on zidovudine (ZDV)/lamivudine (3TC)/nevirapine (NVP) and switched to tenofovir disproxil fumarate (TDF)/3TC/atazanavir/ritonavir (ATV/r) in 2011 due to virological failure. After control of PJP, we switched his HAART to raltegravir (RAL)/darunavir (DRV)/r based on the genotypic resistance report. Within 4 weeks of initiating HAART, he presented with the above mentioned symptoms.

Physical examination was unremarkable except for icterus and tender hepatomegaly. Fundoscopy did not reveal CMV retinitis. He had normocytic normochromic anaemia (Hb: 10.7 g/dl), normal total leucocyte, and differential counts. Liver enzymes were total bilirubin: 9.2 mg/dl (reference value: 0-1 mg/dl), aspartate transaminase (AST): 109 U/L [15–37 U/L], alanine transaminase (ALT): 41 U/L (14–59 U/L), and alkaline phosphatase (ALP): 906 U/L (46–116 U/L). Tests for hepatitis B and hepatitis C were negative.

Hepatomegaly (liver span-18 cm) with grade I-II intrahepatic biliary dilatation with focal parenchymal lesion was seen on abdominal ultrasound. The common bile duct (CBD) was dilated, measuring 14 mm suggestive of possible obstruction at the level of ampulla with no definitive mass or obstructive lesion.

**Figure 1:**
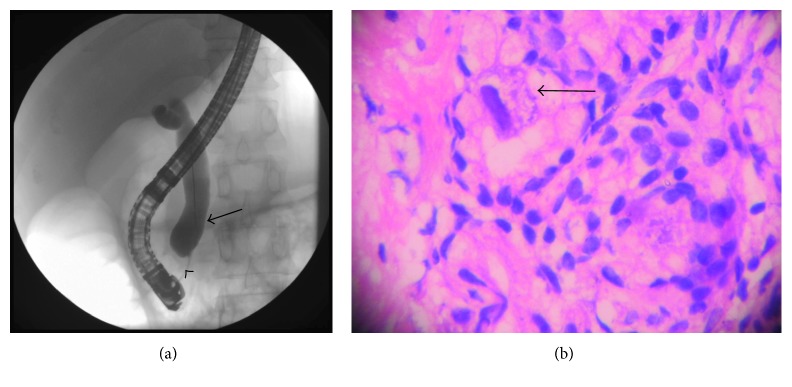
(a) ERCP with cholangiogram showing dilated common bile duct (pointed black arrow) with prepapillary common bile duct stricture (arrow head). (b) Ampullary biopsy (×100) showing submucosal Brunner's glands showing nucleomegaly with eosinophilic intranuclear and basophilic cytoplasmic inclusion bodies (pointed black arrow) of CMV.

An endoscopic retrograde cholangio-pancreatography (ERCP) was performed, which revealed oedematous papilla with stenosis. Cholangiogram showed a dilated CBD with prepapillary CBD stricture. All these features were suggestive of AIDS cholangiopathy (Figure 1(a)).

Wide papillotomy was performed, and a 7 Fr CBD stent was placed for biliary drainage. Bile aspirate was negative for *Cryptosporidium*, *Microsporidium*, *Cyclospora*, and *Mycobacterium*. Brush cytology of the stricture revealed inflammatory cells, pigment-laden macrophages, and several red blood cells with no acid-fast organisms.

Histopathological examination of ampullary biopsy showed focal ulcerations with superficial mucosal necrosis and acute and chronic inflammation in the mucosa and lamina propria. Cells of submucosal Brunner's glands showed nucleomegaly with eosinophilic intranuclear and basophilic cytoplasmic CMV inclusions (Figure 1(b)).

After intervention, the patient had immediate symptomatic relief and did not receive any anti-CMV treatment. Liver enzymes normalised 2 weeks later.

**Figure 2:**
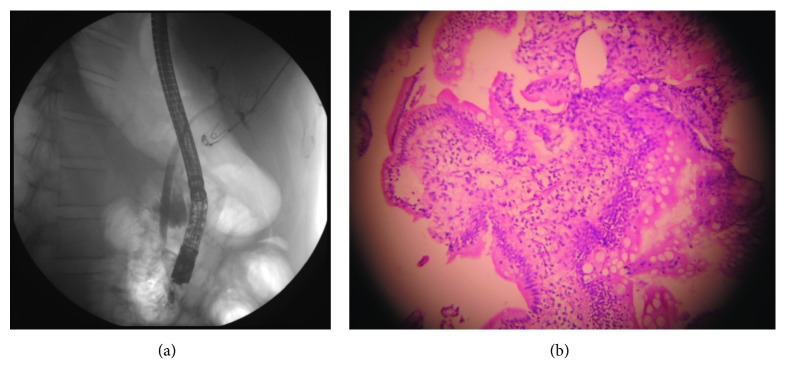
(a) ERCP with cholangiogram with normal CBD and no residual stricture at its lower end. (b) Repeat ampullary biopsy with normal papillary architecture and no CMV inclusion bodies.

After 6 months, CD4 improved to 363 cells/*μ*L with HIV viral load of 423 copies/ml. Repeat ERCP revealed normal cholangiogram with no residual stricture in lower CBD, with normal papilla (Figure 2(a)). Previously placed stent had migrated out. Cells from ampullary biopsies did not reveal intranuclear/cytoplasmic CMV inclusions (Figure 2(b)).

A repeat viral load after 8 months was <50 copies/ml. ART was switched to dolutegravir (DTG)/DRV/r.

## 3. Discussion

We describe here a patient with AIDS cholangiopathy due to CMV occurring within 2 months of switching to effective HAART. Inflammation in the ampullary biopsies and disappearance of CMV inclusion bodies with continuing HAART and without receipt of anti-CMV treatment may suggest this to be unmasking IRIS. To our knowledge, this is the first case report of AIDS cholangiopathy due to CMV IRIS amongst people living with HIV (PLHIV).

IRIS is described as a collection of inflammatory disorders associated with paradoxical worsening of preexisting infectious processes following the initiation of HAART in HIV-infected individuals [4]. In the context of HIV, there are two forms of IRIS: paradoxical IRIS in patients who respond to specific antimicrobial therapy for opportunistic infection (OI) before starting HAART and then have a relapse of OI symptoms after starting HAART, and unmasking IRIS in individuals who present with OI for the first time after HAART is started. Immune reconstitution inflammatory syndrome can occur within 1 week to 4 years, after the initiation of HAART. However, most commonly it occurs within first 3 months [5].

Three reports of CMV-induced duodenal papillitis in AIDS patients and 32 cases of CMV-induced AIDS cholangiopathy have been described till date. Most of these cases have been described prior to the HAART era with some on suboptimal ART such as zidovudine monotherapy [6]. Moreover, few studies have no information on ART use.

AIDS cholangiopathy secondary to IRIS has been reported only once, presented as paradoxical IRIS secondary to *Crytposporidium* [7]. However, no case of CMV IRIS presenting as AIDS cholangiopathy has been reported. CMV-associated IRIS usually manifests as immune recovery uveitis (IRU) or worsening retinitis; preexisting CMV infection may rarely result in IRIS manifesting as pneumonitis, colitis, pancreatitis, or submandibular inflammation [8–10]. We diagnosed IRIS based on the French criteria: (1) major criteria—localised hepatobiliary lesion with evidence of inflammation in ampullary biopsies, and (2) minor criteria—increased CD4 T cell count after HAART and spontaneous resolution of disease with no specific antimicrobial therapy while continuing HAART [11]. Most previous studies of AIDS cholangiopathy due to CMV also documented other end-organ involvement. However, our patient had only localised hepatobiliary involvement.

Anti-CMV treatment does not influence symptoms or cholangiographic abnormalities [2, 12]. The therapy of AIDS cholangiopathy is primarily endoscopic, and the approach varies with the anatomic abnormality. Patients who have abdominal pain, cholangitis, or jaundice associated with papillary stenosis show marked symptomatic relief after sphincterotomy [1, 12]. Isolated or dominant common bile duct strictures can be treated with endoscopic stenting. Sphincterotomy does not help patients with sclerosing cholangitis in the absence of papillary stenosis.

Our patient had prepapillary CBD stricture along with papillary stenosis and papillitis secondary to CMV infection with no evidence of sclerosing cholangitis. *Cryptosporidium*, *Microsporidium*, and *Mycobacterium* were ruled out with relevant stain and histopathology. There was absence of odynophagia or diarrhoea and retinal examination was normal.

Our patient underwent sphincterotomy and CBD stenting with marked improvement in abdominal pain and was continued on HAART; no anti-CMV treatment was initiated. The patient remained asymptomatic on follow-up, with improved appetite; repeat ampullary biopsy showed normal architecture with no evidence of CMV inclusion bodies. In all the previous cases of IRIS secondary to CMV, the patients were treated with either ganciclovir or valganciclovir. Moreover, disappearance of CMV inclusion bodies on histopathology has not been demonstrated in previous CMV cholangiopathy patients.

Raltegravir that was part of the HAART regimen has shown to have in vitro activity against CMV [13]. Moreover, two recent cohort studies suggested a higher risk of IRIS with integrase inhibitor-based HAART (RAL increased risk by 3 times, with no risk with dolutegravir or elvitegravir) than with other regimens and also as an independent risk factor for IRIS in patients with CD4 count of <200/*μ*L and/or with an OI at the time of the HIV diagnosis [14, 15]. Our patient was on integrase inhibitor (RAL) and PI-boosted regimen that may have increased the risk of developing IRIS [16].

Although, we used French criteria to diagnose IRIS, we did not confirm a fall of 1 log in HIV viral load around the time of cholangiopathy. It is also unusual to diagnose CMV end-organ disease in HIV-infected individuals with CD4 count more than 50 cell/*μ*L. Additionally, even in the absence of IRIS, treatment of AIDS cholangiopathy is primarily an endoscopic intervention. However, none of the previous studies have documented histological cure of CMV.

There is a possibility that our patient may have had a CMV OI presenting as AIDS cholangiopathy. However, our patient had CD4 count of 109/*μ*L, and most cases of CMV AIDS cholangiopathy have been described in severely immunocompromised hosts with CD4 <100/*μ*L. However, one case of AIDS cholangiopathy had been reported in HAART-naive patient with CD4 count of >100/*μ*L. Additionally, the diagnosis in this patient was based on serology and high CMV viral load without histological confirmation [17]. In our case, we could not assess CMV DNA quantitative polymerase chain reaction in the plasma.

In conclusion, we report the first case of AIDS cholangiopathy due to CMV presenting as unmasking IRIS. We also demonstrate successful treatment of this condition with endoscopic intervention and continuing HAART. Use of specific anti-CMV treatment may not be warranted in this situation.

## References

[1] Cello J. P. (1989). Acquired immunodeficiency syndrome cholangiopathy: spectrum of disease. *American Journal of Medicine*.

[2] Forbes A., Blanshard C., Gazzard B. (1993). Natural history of AIDS related sclerosing cholangitis: a study of 20 cases. *Gut*.

[3] Yusuf T. E., Baron T. H. (2004). AIDS cholangiopathy. *Current Treatment Options in Gastroenterology*.

[4] Shelburne S. A., Montes M., Hamill R. J. (2006). Immune reconstitution inflammatory syndrome: more answers, more questions. *Journal of Antimicrobial Chemotherapy*.

[5] Williamson P. R., Jarvis J. N., Panackal A. A. (2016). Cryptococcal meningitis: epidemiology, immunology, diagnosis and therapy. *Nature Reviews Neurology*.

[6] Bouche H., Housset C., Dumont J. (1993). AIDS-related cholangitis: diagnostic features and course in 15 patients. *Journal of Hepatology*.

[7] Beauséjour Y., Fortin C., Ghannoum M., Lavergne V. (2011). Immune reconstitution inflammatory syndrome following cryptosporidial cholangitis. *AIDS*.

[8] Miller R. F., Shaw P. J., Williams I. G. (2000). Immune reconstitution CMV pneumonitis. *Sexually Transmitted Infections*.

[9] Jabs D. A., Van Natta M. L, Kempen J. H. (2002). Characteristics of patients with cytomegalovirus retinitis in the era of highly active antiretroviral therapy. *American Journal of Ophthalmology*.

[10] Alukal J., Asif M., Mundada R., McNamee W. B. (2018). Recurrent cytomegalovirus colitis: a rare case of immune reconstitution inflammatory syndrome. *BMJ Case Reports*.

[11] French M. A., Price P., Stone S. F. (2004). Immune restoration disease after antiretroviral therapy. *AIDS*.

[12] Benhamou Y., Caumes E., Gerosa Y. (1993). AIDS-related cholangiopathy. *Digestive Diseases and Sciences*.

[13] Nadal M., Mas P. J., Blanco A. G. (2010). Structure and inhibition of herpesvirus DNA packaging terminase nuclease domain. *Proceedings of the National Academy of Sciences*.

[14] Wijting I., Rokx C., Wit F. Integrase inhibitors are an independent risk factor for IRIS: an athena cohort study.

[15] Dutertre M., Cuzin L., Demonchy E. (2017). Initiation of antiretroviral therapy containing integrase inhibitors increases the risk of IRIS requiring hospitalization. *JAIDS Journal of Acquired Immune Deficiency Syndromes*.

[16] Casado JL., Arrizabalaga J., Montes M. (1999). Incidence and risk factors for developing cytomegalovirus retinitis in HIV-infected patients receiving protease inhibitor therapy. *AIDS*.

[17] Hidalgo-Tenorio C., Blasco-Morente G. (2013). Sclerosing cholangitis by cytomegalovirus in highly active antiretroviral therapy era. *Revista Española de Enfermedades Digestivas*.

